# Quantifying the differences in structure and mechanical response of confectionery products resulting from the baking and extrusion processes

**DOI:** 10.1016/j.jfoodeng.2018.05.039

**Published:** 2018-12

**Authors:** Saba S. Butt, Idris K. Mohammed, Vivek Raghavan, James Osborne, Hugh Powell, Maria N. Charalambides

**Affiliations:** a Mechanical Engineering Department, Imperial College London, South Kensington, London SW7 2AZ, UK; b Nestlé Product Technology Centre Confectionery, Haxby Road, York YO91 1XY, UK

**Keywords:** Baking, Extrusion, Confectionery, Mechanical characterisation, Microstructure

## Abstract

Extrusion has potential advantages over baking in terms of throughput, asset cost and flexibility. However, it is challenging to achieve through extrusion the “light, crispy” texture of a more traditional baked confectionery. This study compares and contrasts for the first time confectionery products produced through these two processes, i.e. baking and extrusion. The microstructural differences are measured using imaging techniques, i.e. Scanning Electron Microscopy (SEM) and X-Ray Tomography (XRT) whereas mechanical characterisation is used to highlight differences in the resulting mechanical properties. Crucial information is presented which shows that the two technologies result in different mechanical properties and microstructures, even if the level of porosity in the two products is kept constant. In addition, confectionery products whether they are produced through baking or extrusion, have irregular geometries. The latter makes mechanical characterisation a real challenge. Therefore this study also presents rigorous methods for measuring true mechanical properties such that meaningful and valid comparisons may be made. The accuracy of the chosen methodologies is verified through experiments using flat and tubular extruded geometries as well as testing the products in various directions. It was concluded that the manufacturing method and, in the case of extrusion, the initial moisture content influences the microstructure and mechanics of confectionery products, both of which have an impact on consumer sensory perception.

## Highlights

• Extrusion has advantages over baking but it is hard to achieve a light/crispy texture.
• Products made via the two technologies are compared microstructurally and mechanically.
• Rigorous methods for irregular product geometries were developed for valid comparisons.
• Our data are required as inputs to computational predictive models of the bulk response.
• Quantification of differences paves the way for optimising extrusion processes.

## 1 Introduction

Oven baked wafers are intermediate components used in the manufacture of several popular confectionery products and have been manufactured and marketed successfully for decades (Sundara, 2012). A more recent method of producing similar lightweight and crispy products is the extrusion process. Extrusion gives many advantages over the conventional cooking processes in terms of throughput, asset cost and flexibility; it is a continuous process with the flexibility of on-line process adjustments for achieving the desired product characteristics (Karwe, 2008). However, attaining a similar ‘light’, crispy texture through extrusion as in a traditional baked confectionery wafer is problematic. This study aims to understand why products produced through these two processes are different, quantify any differences both microstructurally and mechanically and therefore pave the way for optimising extruded processes and products.

Consumers base their perception and appreciation of acceptable foods on characteristics such as crispness or crunchiness of the food. A review by Luyten et al. (Luyten et al, 2004) reported the absence of an officially accepted definition and measurement of crispness, or the characterisation of properties of crispy foods. However, a large proportion of experiments and studies (Sandoval et al., 2008, Arimi et al., 2010, Roudaut et al., 1998, Ashby, 2006) in this field appear to use the fundamental mechanical properties, namely Young's Modulus and fracture stresses, as a means to characterise and compare the mechanical properties of crispy food products. Typical mechanical tests that are popular in food testing include tensile, compression, bending and puncture tests (Duizer, 2001, Duizer, 2003). It is a general consensus that both ‘crispy’ and ‘crunchy’ sensations relate to the fracture properties of food materials (Luyten et al, 2004). It is suggested that ‘crunchy’ foods exhibit a complex fracture behaviour correlating to frequent drops in the force during compression or indentation loading, i.e. frequent fracture events. These mechanical ‘signatures’ (or indeed their associated acoustic emission traces) have also been analysed so that their ‘ruggedness’ was quantified in numerical terms using Fast Fourier transform analysis as well as fractal analysis (Roudaut et al., 2002, Pamies et al., 2000) in an effort to draw correlations to sensorial measurements. The presence of such frequent fracture events implies that the morphology plays an important role in the crunchiness of the material as fracture events are expected to correlate to fracture of ‘layers’ in the microstructure, typical of the fracture behaviour of cellular materials (Luyten et al, 2004).

The morphology of cellular materials can be characterised by the porosity, relative density, the size and shape of the cells and their distribution in the microstructure as well as the amount of solid material present in the cell faces and edges (Ashby, 2006). The latter is used to classify the foam as an ‘open’ or ‘closed’ celled structure and affects the interconnectivity of the cells. Microstructure in this study will refer to the arrangements of the pores and cell walls, not the supramolecular level of the solid material. A number of authors have developed analytical models in order to determine the solid material properties of the foams. These models treat the porous microstructure as complicated shapes, for example, Chen and Lake developed a model for closed cell foams based on tetrakaidecahedral unit cell shape (Butt, 2016), the Halpin-Tsai model is a commonly used model for composites (Williams, 1980), the Christensen model considers open celled foams as a three dimensional network of struts (Christensen, 1986) and the Gibson and Ashby model treats the foam as an array of simple cubic cells (Ashby, 2006).

Therefore, mechanical (texture) properties heavily influence the quality of a food product as perceived by the consumer. Now, in the engineering field of mechanics of materials, it is a well-known fact that these macroscopic properties are affected by the food structural organisation at the smaller length scale (Clarke and Eberhardt, 2002). Hence, it is of great importance and interest to study the food structure at the microscopic level and determine its associated effect on the properties at the macroscopic level. The microstructure of food foams can give crucial information in combination with the results from the mechanical tests (Mohammed et al., 2013a, Agbisit et al., 2007). Agbisit et al. (2007) suggested that there is a moderate to strong association of the mechanical properties of the food foams with their cellular structure. The Young's modulus of cellular foams can be correlated to cell dimensions and the cell wall thickness (Gao and Tan, 1996). It is worth noting here that the porous nature of food foams necessitates the definition of two densities; one of the solid material and another of the bulk foam. The solid density, *ρ*_*s*_, and bulk foam density, ρ*, can be used to determine the relative density of the foam (ratio of air space to solid material) which can in turn be related to the foam porosity (Nussinovitch, 2005).

Some literature relates to baked bread, biscuit, wafer and extruded foods (Agbisit et al., 2007, Chanvier et al., 2013, Yven et al., 2010, Chevallier et al., 2014, Livings et al., 1997), however, this study is the first to investigate the products made via these two processes in comparison with each other; most importantly all the materials in this investigation were produced using manufacturing facilities at one location and ingredients sourced from the same place. This can give crucial information about whether the two technologies result in different types of products they can produce or whether they are producing an end product of similar mechanical properties and therefore sensory and textural attributes. It also aims to expand the knowledge and understanding of food foams and their link to sensory properties, an area with a great deal of scope for further research.

Lastly, the irregular and complex geometry of confectionery extruded and baked products require rigorous experimental characterisation methodologies; this study will therefore also highlight such methods to enable valid comparisons between the mechanical response of several products to be made. Such rigorous methods are generally lacking in the food research literature, with geometry dependent tests based on Texture Profile Analysis (TPA) often used in the form of penetration and puncture experiments which though they may at best be able to rank various materials or products, they are not able to result in fundamental, geometry independent, mechanical properties. For samples of highly variable geometry which often is the case with food products, the accuracy and meaning of the resulting TPA data is doubtful. Finally, rigorous fundamental mechanical properties are also needed as inputs to computational predictive models of other downstream processes such as cutting, packaging or indeed of the food oral process.

## 2 Materials and methods

### 2.1 Samples

Because of the inherent nature of the two manufacturing processes, baking within hot plates produces flat sheets whereas extrusion often leads to cylindrical shapes, as is the case in this study. Therefore, in an effort to study the effect of the product's geometry, the extruded shapes were rolled upon exit of the die to produce a flatter shape (called a ‘flatbread’ in this work) to provide a more direct comparison with the baked sheets. Additionally, the water content in the pre-extrusion mix was also studied as this could affect the density of the extruded shape, though it is not easy to predict the outcome. This is because expansion of starchy melts during the extrusion process is a complex phenomenon; several parameters such as temperature, moisture content and die geometry affect the different mechanisms related to expansion, i.e. bubble nucleation, growth, coalescence shrinkage and finally setting (Kristiawan et al., 2016). As it will later be discussed, in the current study the higher moisture content led to a higher density product and vice versa. Hence the effect of water content is studied by comparing ‘standard’ (SD) and ‘high density’ (HD) tubes corresponding to lower and higher water contents respectively.

**Fig. 1:**
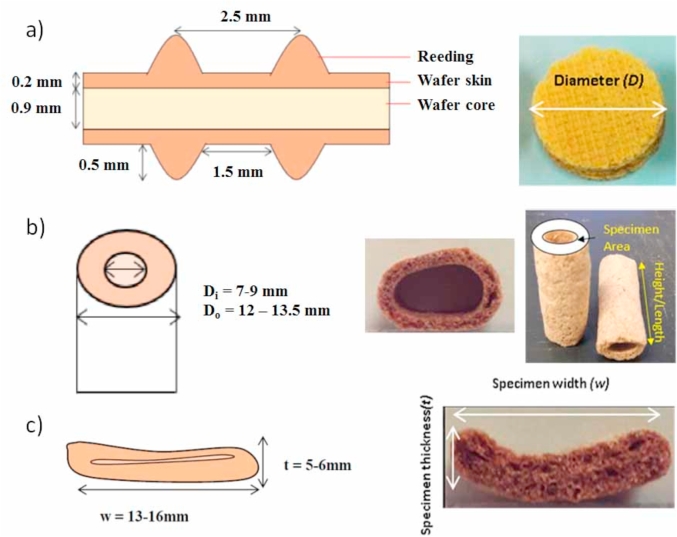
Schematic and photos of (a) Baked wafer (b) Extruded tube (c) Extruded flatbread [schematics not drawn to scale].

Therefore the materials required for this study consisted of four samples: a standard density (SD) extruded tube, a high density (HD) extruded tube, a high density (HD) extruded flatbread (all three produced via the same extrusion process), and a wafer product made via the baking process (Fig. 1). All samples were provided by Nestlé Product Technology Centre Confectionery, York and their formulations are summarised in Table 1. The wafer batter ingredients consist primarily of wheat flour and water while the mixture used for the extrusion process is made by varying the quantities of the same ingredients and adding cocoa powder, sugar and starch.

**Table 1:** Formulations of baked (wafer) and extruded (SD Tube and HD Tube) samples. Flatbread had the same formulation as the HD Tube.

Ingredients (% w/w)	Baked Wafer	Extruded SD Tube	Extruded HD Tube
Wheat flour (white)	50.5	19.1	18.7
Water	48.3	2	4
Corn starch (native)	–	32.3	31.7
Corn starch (modified)	–	19.3	18.9
Sugar (white)	–	20.6	20.2
Alkalised cocoa powder	–	3.9	3.8
Vegetable Oil	0.7	–	–
Minor ingredients	0.5	2.8	2.7

In the baking process, liquid batter is spread and then baked between in a single plate Haas oven (Haas Food Equipment GmbH, Austria). The oven plates have engravings, known as ‘reedings,’ which allow the batter to spread evenly when the hot plates are closed and also allows ease of removal by preventing the batter from sticking to the plates. During the baking process most of the moisture evaporates, resulting in a porous cellular structure which is lightweight and crisp. The wafers were baked at a temperature of 152 °C and for a duration of 110s. The three extruded samples (SD and HD tubes, flatbreads) are made by passing the mixture through a Clextral BC45 twin screw extruder (Clextral, France) at high pressure and temperature where the semi cooked material exits the die as a cylindrical product. The extruded material takes between 30 and 60 s to solidify. During this period, the cylindrical extruded material can be pressed under rolling pins to achieve an extruded ‘flatbread’ product. The densities of the extruded products are varied by changing the level of water addition in the original mix. The HD tube and flatbreads were made from the exact same formulation.

The baked wafers were in the form of rectangular sheets with a maximum peak-to-peak thickness of 2.3 mm, a peak-to-peak length of 2.5 mm and a length of 1.5 mm between the troughs (see Fig. 1a). The extruded products were in the shape of hollow cylindrical tubes with outer diameter *(D*_*o*_*)* ranging between 12 and 13.5 mm, and inner diameter *(D*_*i*_*)* between 7 and 9 mm and as extruded flatbreads of width *(w)* between 13 and 16 mm and thickness *(t)* of 5–6 mm (see Fig. 1b and c).

All samples were stored under controlled conditions at temperatures between 20 and 25 °C and relative humidity between 20 and 30% for five days before testing. This conditioning would result in a moisture content in the tested samples of approximately 4%. The relative humidity was controlled by keeping the samples in a sealed ventilated chamber over potassium acetate salt. These salt crystals control the relative humidity by absorbing or releasing moisture into the environment. It was important to keep the samples in a controlled and regulated environment as the products have a tendency to absorb moisture which can cause the material properties to change. Samples were prepared according to the dimensions required for testing before storing them in the storage chamber in order to minimise the time that the samples were kept out of the controlled environment before testing.

### 2.2 Microstructural characterisation

The characterisation of the internal microstructure of the products was performed by imaging the cross sectional area using a Hitachi S-3400 Scanning Electron Microscope (SEM). The SEM operated at a voltage of 15 kV in either the secondary electron or the backscattered electron mode. In order to obtain high quality images, the samples were coated with a thin layer of gold to achieve a conductive surface. Specimens of 5–10 mm in length were cut from the baked wafer sheets while the extruded products had a fixed outer diameter. The samples were placed on the SEM stage so that the cross section of the sample faced the microscope lens. SEM images were then imported into image analysis software, ImageJ, in order to quantify the microstructure in terms of pore sizes and cell wall thickness.

X-Ray Tomography (XRT) was also used to characterise the extruded products, (SD tube and HD tube only) and baked wafer. Unlike the SEM method mentioned above, XRT allows for the measurements to be taken across the volume of the product. An area of both products was scanned under a cabinet cone-beam microCT scanner (SCANCO Medical AG, Bruttisellen, Switzerland) in order to produce a stack of image slices of the cross section of the products. The SD tube was scanned with a μCT 50 scanner with a voltage of 55 kVp, 4.4 μm voxel size and an image stack of 500 slices and the HD tube was scanned with a μCT 100, Voltage 70 kVp, 6.6 μm voxel size and an image stack of 500 slices. A fine resolution was selected as it can heavily affect the determination of quantitative measurements such as pore sizes and cell wall thickness (Chevallier et al., 2014). The image stack produced by the XRT scan was used to create a 3D volume of the microstructure using the software Avizo (v6.3.0, Visual Sciences Group, Burlington, MA, USA). The reconstructed 3D volume was then used to determine the porous volume fraction, *φ*, of the products and as a result the relative density, *ρ*_*relative*_, of the foam. The relative density is the most significant structural characteristic of a cellular solid and it related to the porosity, *φ*, via Equation (1) (Nussinovitch, 2005):

(1) $$\phi =1-\rho_{relative}$$

ImageJ analysis tools were subsequently used to compute the distribution of wall thickness and pore diameter to accurately characterise the microstructure from the raw XRT scans of the products. The XRT image stack allows for the cell wall and pore sizes to be measured throughout the volume of the sample unlike the SEM images which only allow analysis on a single 2D plane.

In order to validate the accuracy of the *φ* and *ρ*_*relative*_ measurements obtained from the XRT analysis, the bulk foam density, *ρ*_*foam*_, and solid density, *ρ*_*solid*_, were determined experimentally via two density measurements techniques, the spheriglass-beads displacement and helium pycnometry respectively (Paul, 2001). Spheriglass-beads of diameter of 0.35 mm, were poured in a volumetric cylinder and the corresponding volume was recorded. The mass of the food sample was measured and submerged into the glass-beads in the cylinder. The displaced volume of the beads containing the sample was measured in order to calculate the bulk density *ρ*_*foam*_. A micrometric AccuPyc 1330 pycnometer was used to determine the solid density *(ρ*_*solid*_*)* of the products. The samples were weighed and placed in the sample chamber which was sealed and filled with helium gas. The solid density was found by measuring the amount of the displaced gas in the chamber. The *ρ*_*foam*_ and *ρ*_*solid*_ measurements were then used to calculate an independent estimate of *ρ*_*relative*_ through:

(2) $$\rho_{relative}=\frac{\rho_{foam}}{\rho_{solid}}$$

### 2.3 Mechanical characterisation

Uniaxial compression tests were performed using a universal Zwick Roell 1.0 (Zwick Testing Machines Ltd., UK) testing machine with a 1 kN load cell. Compression tests have a key advantage over tensile tests, as they do not require the sample to be gripped which might be difficult to achieve without slip or specimen breakage and are also the appropriate choice due to its relevance to the mastication process (Mohammed et al., 2013a). All tests were conducted at ambient conditions of 20 °C room temperature and 50% relative humidity.

**Fig. 2:**
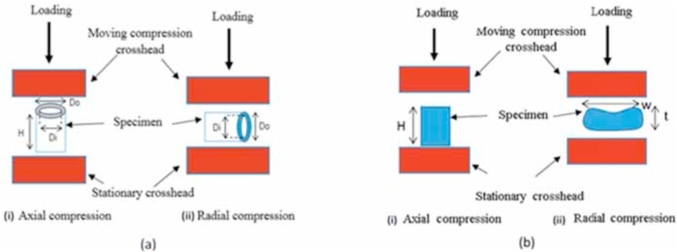
Orientation of the extruded (a) tube and (b) flatbread under (i) axial and (ii) radial compressive loading.

For compression tests, the wafer samples were cut into circular specimens of 40 mm diameter using a boring tool and had a height *H* of 2.3 mm. The cross section of the SD and HD extruded tubes was constrained by the initial dimensions of the outer diameter, *D*_*o*_, and inner diameter, *D*_*i*_. The extruded specimens were cut using a sharp blade to a height, *H*, which was chosen such that the aspect ratio *H/D*_*o*_ was close to 1. It was difficult to prepare shorter samples as the samples were brittle and had a tendency to fracture during cutting. The cylindrical geometry of the extruded product allowed for it to be loaded in two different directions, namely axial compression and radial compression, as shown in Fig. 2. The dimensions of the samples used for radial compression were the same as specified above. Similarly, the extruded flatbreads, width *(w)* 16–17 mm, height *(H)* 16–17 mm and thickness *(t)* 5–6 mm, were also loaded in the two directions that the tubes were tested in i.e. axial and ‘radial’ compression. The term ‘radial’ here refers to the direction before the extruded tube was rolled to its final flatbread shape. In contrast, the baked wafer's geometry allowed uniaxial compression only in a single direction, parallel to its smallest dimension.

The raw load *(F)*-displacement *(δ)* data from the axial compression tests of the products were converted into stress *(σ)* values via Equation (3) for the extruded tubes, Equation (4) for the HD flatbread and Equation (5) for the wafer. The strain *(ɛ)* was calculated using Equation (6) for all the products. *D*_*o*_ and *D*_*i*_ were used to calculate the cross-sectional area of the tubes. The cross section of the extruded flatbread was treated as a rectangle to calculate the area, where *w* is the width of the flatbread and *t* is the thickness as shown in Fig. 2b. At least three measurements of all dimensions were taken at different locations on the samples before testing and average dimensions were used in the calculation of stress and strain.

(3) $$\sigma_{tubes}=\frac{F}{\pi \left({\left(\frac{D_{o}}{2}\right)}^{2}-{\left(\left(\frac{D_{i}}{2}\right)\right)}^{2}\right)}$$

(4) $$\sigma_{flatbread}=\frac{F}{w\times t}$$

(5) $$\sigma_{wafer}=\frac{F}{\pi {\left(\frac{D}{2}\right)}^{2}}$$

(6) $$\varepsilon =\frac{\delta }{H}$$

The axial modulus *(E*_*axial*_*)* was calculated from the initial linear region of the stress-strain graph. Equations (3), (4) assume regular annular and rectangular geometries for the extruded tube and flatbread respectively and taking average readings for each sample for *D*_*o*_, *D*_*i*_, *w* and *t*; this was to simplify the calculation. The error introduced due to this assumption was checked via comparing the actual cross sectional area to the regular (annular and rectangular) geometry area for a few samples and it was found to be negligible.

For the radial compression tests, the radial modulus *(E*_*radial*_*)* was calculated using the analytical solution for the compression of a thin ring (Williams, 1980):

(7) $$E_{radial}=\frac{F}{\delta }\left(\frac{\pi }{4}-\frac{2}{\pi }\right)\frac{a^{3}}{H{\left(\frac{D_{o}-D_{i}}{2}\right)}^{3}}$$

where *a* is the radius of the thin ring and for this present study is assumed to be the average of the outer and inner radii of the tube. Since the ratio of the outer to inner diameter of the tubes was approximately equal to two, and thus was closer to a thick ring rather than a thin ring, a finite element model of the radial compression (described in Section 3.3.2) was performed as to verify the validity of the ring analysis. A numerical analysis was also performed on the HD flatbread to determine the *E*_*radial*_ since an analytical solution was not possible due to its complex shape. The FE models consisted of the product geometry between two rigid plates, thus mimicking the experimental setup.

**Fig. 3:**
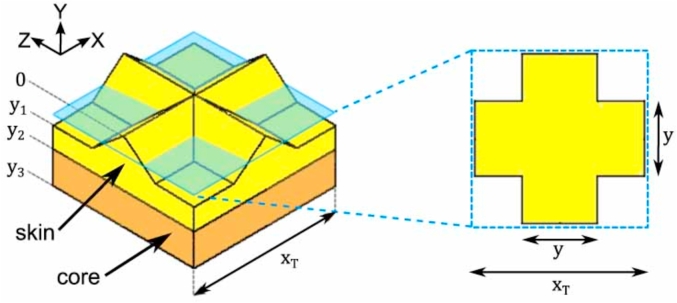
Cross-section of an element of the actual geometry of the baked wafer with measurements required for the analytical model for compression (Pamies et al., 2000).

The reedings present in the wafer sheet make the calculation of the wafer modulus more complicated than that of the extruded tubes. An analytical solution which takes into account the geometry of the reedings (Mohammed et al., 2013a), was therefore used to calculate the moduli values of the baked wafer in compression. In this work the structure of the wafer was simplified such that the skin and core regions were assumed to be the same, homogeneous material with a compressive modulus, *E*_*wafer*_. Equation (8) was used to calculate the compressive modulus of the baked wafer where *x*_*T*_*, y, y*_*1*_ and *y*_*3*_ are various features in a unit cell of the wafer geometry as shown in Fig. 3 and were measured from the SEM scans of the microstructure of the baked wafer. The measured values are listed in Table 2. *F/δ* is the stiffness gradient obtained from the load-displacement curves of the compression tests.

(8) $$E_{wafer}=\frac{F}{\delta }\left[\int_{y_{1}}^{y_{3}}\frac{1}{{\left(x_{T}\right)}^{2}}\text{d}y+\int_{0}^{y_{1}}\frac{1}{4y\left(x_{T}-y\right)}\text{d}y\right]$$

**Table 2:** Sample dimensions of all the products used in compression tests.

Product type	Dimensions [mm]
Baked wafer sheet	*D* = 40
	*H* = 2.3
	*x*_*T*_ = 2.5
	*y*_*1*_ = 0.5
	*y*_*2*_ = 0.2
	*y*_*3*_ = 0.45
Standard density (SD) extruded tube	*D*_*o*_ = 12.9 ± 0.6
	*D*_*i*_ = 7.5 ± 0.5
	*H* = 15
High density (HD) extruded tube	*D*_*o*_ = 12.5 ± 0.4
	*D*_*i*_ = 8.5 ± 0.3
	*H* = 15
High density (HD) extruded flatbread	*w =* 16–17
	*H = t =* 5–7

The dimensions of all the samples, baked and extruded, prepared for compression tests are listed in Table 2.

## 3 Results

### 3.1 Microstructure characterisation

Table 3 shows the results for the densities and porosities of the four products. The errors shown are standard deviations derived from at least five test repeats for each sample. The values of *ρ*_*foam*_, *ρ*_*solid*_ (determined from the spheriglass-beads displacement and helium pycnometry respectively) and *ρ*_*relative*_ of the baked wafer and the SD tube were found to be similar. The solid density, *ρ*_*solid*_, of all the extruded products was similar, with an average value of 1.53 g/cm^3^, and somewhat higher than the corresponding value of the wafer which was 1.40 g/cm^3^. This confirms that all the extruded products are made from a similar material. The HD extruded tube and flatbread were found to have a higher foam density, *ρ*_*foam*_, compared to the SD tube which resulted in a higher relative density, *ρ*_***relative***_. Next, the *ρ*_*relative*_ values are used with Equation (1) to calculate the foam porosity, *φ*. It is seen that *φ* of the SD tube and baked wafer products was found to be very similar at around 78%. The HD tube and flatbread had a lower porosity by an amount of 11% and 16% respectively, when compared to the SD tube which is due to a higher flour:water ratio present in the SD extruded products. In addition, *ρ*_*foam*_ for the flatbread is somewhat higher than the corresponding value of the HD extruded tube and this is thought to be due to the loss of some porosity during the rolling process of the HD tube straight after extrusion. Finally the porosity values, *φ*, obtained from the XRT images are compared to the ones obtained from the density measurements and the two are found to be very close to each other, giving further confidence in the accuracy of the results. As the wafer and SD tube had the same porosity, these two products will be compared against each other below in order to investigate the effect of processing on the product structure.

**Table 3:** Density measurement results for extruded products and baked wafer.

	Baked wafer (skin & core)	Standard density (SD) extruded tube	High density (HD) extruded tube	High density (HD) extruded flatbread
*ρ*_*foam*_ (g/cm^3^)	0.31 ± 0.03	0.34 ± 0.01	0.50 ± 0.11	0.58 ± 0.18
*ρ*_*solid*_ (g/cm^3^)	1.40 ± 0.02	1.52 ± 0.003	1.53 ± 0.03	1.53 ± 0.01
*ρ*_*relative*_	0.22 ± 0.04	0.22 ± 0.01	0.33 ± 0.07	0.38 ± 0.12
*φ* (%) from densities	77.7 ± 4.0	78.0 ± 1.0	67.1 ± 7.0	62.0 ± 12.0
*φ* (%) from XRT	79.0	78.0	65.0	–

**Fig. 4:**
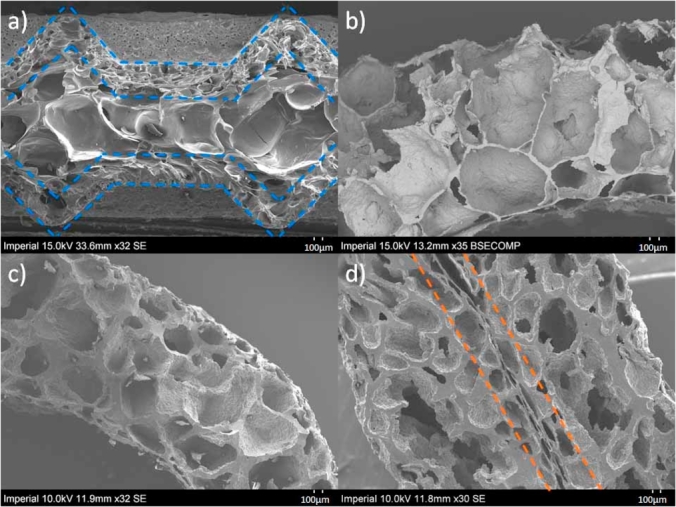
Typical cross-sections a) Baked Wafer b) Standard density (SD) extruded tube c) High density (HD) extruded tube and d) HD extruded flatbread obtained from the SEM.

**Fig. 5:**
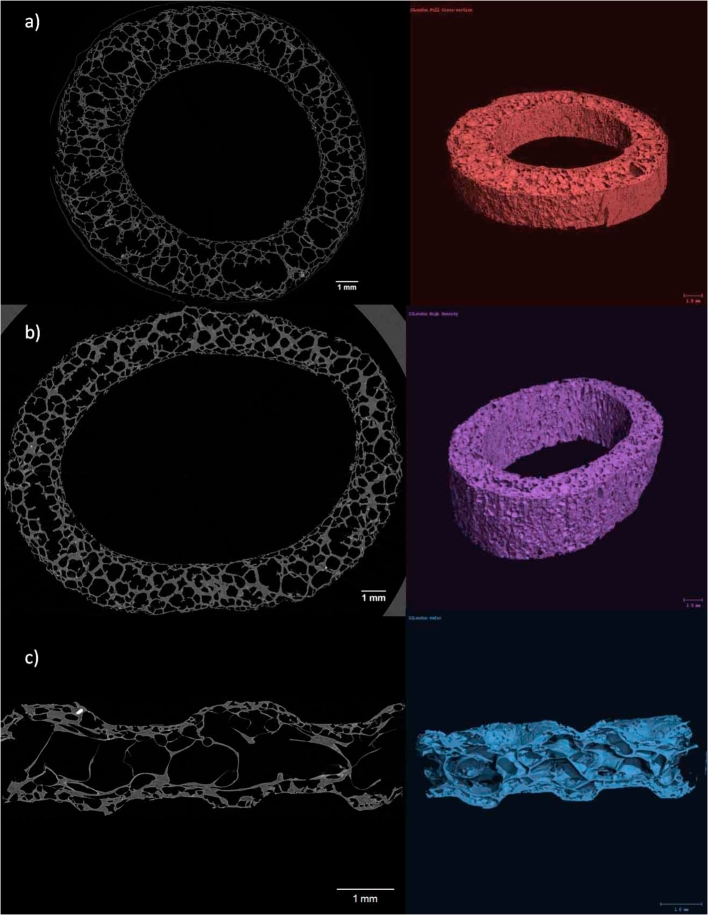
–Typical cross sections and 3D renderings of a) Standard density (SD) extruded tube b) High density (HD) extruded tube and c) baked wafer obtained from XRT scans.

Fig. 4a–d show the cross-sections of the baked wafer and extruded products obtained from the SEM. The XRT scans obtained for the baked wafer and extruded tubes are shown in Fig. 5a–c. The image analysis software ImageJ was used to compute graphs of the distribution of wall thickness and pore diameter to accurately characterise the microstructure from the raw XRT scans of the products. XRT scans were not performed on HD flatbreads. The results from both SEM and XRT analyses are presented in Table 4, Table 5 respectively.

**Table 4:** SEM analysis of microstructure of the baked wafer and extruded products.

Product type	Cell wall thickness range (μm)	Average cell wall thickness (μm)	Pore size range (μm)	Average pore size (μm)
Baked wafer (core)	5–25	19	50–1100	600
Baked wafer (skin)	10–200	87	50–150	83
Standard density (SD) extruded tube	10–220	51	30–980	296
High density (HD) extruded tube	14–220	65	51–1263	422
High density (HD) extruded flatbread	12–255	61	54–1121	345

**Table 5:** XRT analysis of the microstructure of the extruded tubes.

Product type	Cell wall thickness range (μm)	Average cell wall thickness (μm)	Pore size range (μm)	Average pore size (μm)
Baked wafer (core)	7–124	46	19–1280	233
Baked wafer (skin)	7–188	78	19–681	115
Standard density (SD) extruded tube	11–440	88	11–1100	100
High density (HD) extruded tube	10–772	173	16–1100	228

From Fig. 4, Fig. 5, a cellular foam structure was observed for all the products. The SEM images of the baked wafer (see Fig. 4a) showed a denser skin region near the outer edges of the wafer and a core region near the centre of the cross section (indicated by the dotted red lines in Fig. 4a), which consisted of larger pores and thinner cell walls as compared to the skin. This observation is in agreement with the findings stated by Mohammed et al., 2013a, Mohammed et al., 2013b, and Mohammed (2001). In comparison, the extruded tubes (Fig. 4b and c) had more homogenous cellular structures with a non-uniform thickness around the annulus. The extruded flatbread (Fig. 4d) presented a dense region through the centre which was a result of the extruded tube being compressed straight after the extrusion process. This caused the inner edges of the tube to align together to form a ‘lip’ like region through the middle (indicated by the dotted red lines in Fig. 4d) and the pores on the outer edges also seemed to have been compressed to form denser skins on the outer edges of the flatbread.

By comparing the data shown in Table 4, Table 5, the pore diameter and cell wall thickness ranges and averages measured from SEM and XRT for the wafer and extruded tubes were found to be roughly in the same order of magnitude. A quantitative SEM analysis is restricted to a few random cross-sections of the samples, whereas the XRT analysis takes measurements of hundreds of cross-sections throughout the whole volume and thus more statistically accurate. XRT data was not available for the flatbread but from Table 4 it may be concluded that the two HD products had very similar microstructures.

**Fig. 6:**
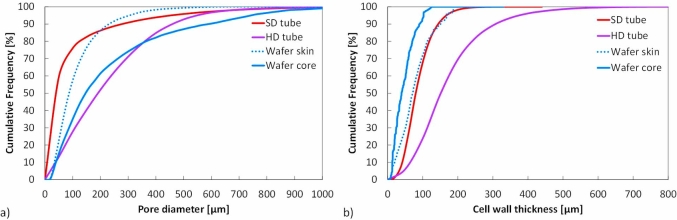
(a) Pore size distribution and (b) Cell wall thickness comparison of high density extruded tube and standard extruded tube.

The XRT data was next used to generate distribution graphs of the pore size and the cell wall thickness, as shown in Fig. 6, using each 2D image cross-section in the image stacks of the baked wafer and extruded tubes. The range and average of these distributions are also given in Table 5. The pore size distribution is the abundance of each pore size in a representative volume of a material (Nimmo, 2004). The pore diameter range was found to be similar in both extruded tubes and the baked wafer core (Table 5), however the distribution in the diameters was very different (Fig. 6a). The SD tube (as well as the wafer skin) had much higher proportion of cells below a diameter of 200 μm (86%), as compared to the wafer core and HD tube which only had 61% and 52% respectively below 200 μm. This was also reflected in the higher average pore size in the wafer core (233 μm) and the HD tube (228 μm) when compared to the wafer skin (115 μm) and the SD tube (100 μm) as shown in Table 5. Between the two extruded tubes, the HD tube was also found to have thicker cell walls with a higher mean value, i.e. 173 μm, compared to 88 μm for the SD tube). As seen in Figs. 6b and 24% of the cell walls in the HD tube were below 100 μm whereas the corresponding value was 68% in the SD tube. The average cell wall thickness of the wafer core (46 μm) was less than that of the skin (78 μm), with 90% of the cell walls in the skin being below 150 μm as compared to the core of 90 μm. The cell wall thickness average and distribution curve of the baked wafer skin was very similar to that of the SD tube, although the latter possessed a larger range of thicknesses. The difference in the pore size and cell wall thickness distributions between the products is a consequence of the different processing conditions during baking and extrusion as well as the initial moisture content.

To conclude, the manufacturing process (baked or extrusion) leads to different microstructures even if the relative density is kept similar as was the case between the wafer and the SD tube. During baking, the product gets heated first in the proximity of the baking plates thus forming the two skins; this leaves the middle of the product still fluid and hence able to form a core with larger pores and smaller wall thickness than the wafer skins and the SD tube. In addition, by comparing the SD and HD tubes, it is seen that a lower water addition in the mix leads to a lower density product with larger pores and cell walls.

### 3.2 Mechanical characterisation

A summary of all the mechanical test results is presented in Table 6. The individual test data will be discussed separately in the following subsections.

**Table 6:** Mechanical properties of extruded products and baked wafer.

Material property	Baked Wafer Modulus (MPa)	Standard Extruded (SD) Tube (MPa)	High density (HD) Extruded Tube (MPa)	High density (HD) Extruded Flatbread (MPa)
Axial compression modulus, *E*_*axial*_	4.7 ± 0.4	44.6 ± 9.4	103.7 ± 11.4	136.6 ± 62.2
Fracture stress in axial compression, *σ*_*axial*_	0.5 ± 0.01	0.6 ± 0.1	1.2 ± 0.4	4.0 ± 1.6
Radial compression modulus, *E*_*radial*_ (analytical)	–	36.9 ± 8.8	114.1 ± 17.2	
Radial compression modulus, *E*_*radial*_ (numerical)	–	49 ± 12	132 ± 20	63 ± 18.5
Fracture stress in radial compression, *σ*_*radial*_ (numerical)		1.0 ± 0.2	2.1 ± 0.2	

#### 3.2.1 Axial compression of extruded products and baked wafer

The axial compressive Young's Moduli (*E*_*axial*_) of all the products were found to be independent of the test speed over three orders of magnitude (Butt, 2016) and therefore the tests were conducted at the speed of 1 mm/min for the baked wafer and extruded flatbreads and at 10 mm/min for the SD and HD extruded tubes. These speeds were selected to ensure the safety of the testing rig and were based on the dimensions of the products, for example, a lower speed was selected for the baked wafer due to its very small sample height (2.3 mm).

**Fig. 7:**
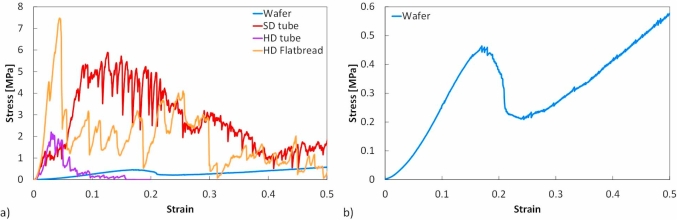
Comparison of a) typical stress-strain curve of baked wafer and extruded products under axial compression b) typical stress-strain curve of the wafer plotted using a smaller stress scale for clarity.

Five to seven repeats were conducted for each product and a comparison between typical stress-strain responses of the products under uni-axial compressive loading is shown in Fig. 7a. *E*_*axial*_ was calculated from the initial linear elastic region for each of the products. The axial compressive moduli of SD tube, HD tube and HD flatbread were found to be 44.6 ± 9.4 MPa, 103.7 ± 11.4 and 136.6 ± 62.2 MPa, respectively while the average fracture stresses (*σ*_*axial*_) under axial compression were found to be, 0.6 ± 0.1 MPa, 1.2 ± 0.4 and 4.0 ± 0.4 MPa respectively. Using Equation (8) derived from an analytical model for calculating the modulus by taking into consideration the actual geometry of the wafers (Mohammed et al., 2013a), the homogenous compressive modulus for the baked wafer was found to be 4.7 ± 0.4 MPa whilst the fracture stress was calculated as 0.5 ± 0.01 MPa from Equation (5). A previous study (Mohammed et al., 2013a) performed on similar baked wafers but a different batch, reported a homogeneous compressive modulus value of 3.9 MPa which is in reasonable agreement with the value stated here.

In compression, the stress-strain curves of all products showed a typical compressive response of brittle foams with an initial linear region followed by an initial fracture and a jagged plateau. The stress-strain response of the extruded products displayed a large amount of jaggedness throughout the test, correlating to more frequent fracture events as compared to the baked wafer. In comparison, the baked wafer had one major fracture event, signifying the initial collapse within the core, followed by minor fractures of the remaining cell walls until densification (see Fig. 7b).

#### 3.2.2 Radial compression of extruded products

**Fig. 8:**
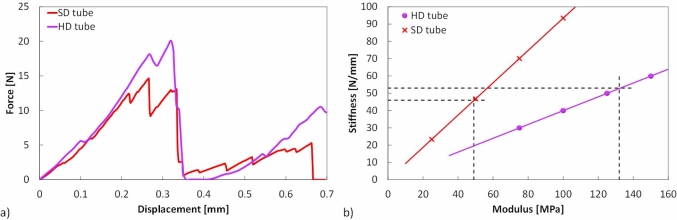
(a) Typical experimental Load-deflection data from radial compression of the extruded tubes and (b) FEA results for estimating E_radial_ of SD and HD extruded tubes.

Five to seven replicate radial compression tests were performed for each extruded product. As shown in Fig. 8, an initial linear region is observed, from which the stiffness was obtained. The force then falls to almost zero as the tube fractures into two halves, with a vertical fracture extending through the thickness of the annulus from the inner to the outer diameter at the points of contact with the plates. The experimental radial compression load-deflection data for of the SD and HD extruded tubes (Fig. 8a) were used to calculate the radial compressive modulus, *E*_*radial*_, (Fig. 8b) using Equation (7). This radial compression analysis is useful as it is representative of the strength of the product in the direction in which it is likely to be consumed. The *E*_*radial*_ values of the SD tube and HD tube were found to be 36.9 ± 8.8 MPa and 114.1 ± 17.2 MPa respectively.

**Fig. 9:**
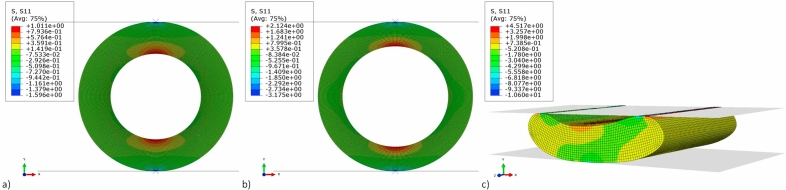
Finite element horizontal (S11) stress contour plots of the compressed extruded products (a) SD tube (b) HD tube (c) HD flatbread. Units are in MPa. Displacements for the SD and HD tubes are set equal to the fracture displacements shown in Fig. 8(a). Flatbread displacement was set equal to 2 mm.

However, the derivation of Equation (7) assumes a thin ring, ie. similar outer and inner diameters, which is not the case of the extruded tubes in this work. Thus, as already mentioned in section 2.3, a radial compression finite element model was developed, using the commercial finite element software, Abaqus (Simulia, 2013), in order to numerically determine *E*_*radial*_ and check the validity of the analytically determined *E*_*radial*_ values. The SD and HD tube geometries were modelled as 2D annuli with their respective outer, *D*_*o*_, and inner, *D*_*i*_, diameters and out of plane lengths, *H* (Table 2). Two 2D analytical rigid parts were used to create the compression plates, with the lower compression plate constrained in all directions and a displacement in the radial loading direction of the tube applied to the upper compression plate in order to simulate the compressive deformation. A contact definition was applied between the outer surface of the virtual extruded product and the rigid bodies with the interaction assumed to be a hard contact (no penetration) and frictionless. A mesh convergence study was conducted with linear 2D linear plane strain elements in order to determine the optimum mesh size for both of the tube geometries. Deformed stress contour plots of the SD and HD tube FE models are shown in Fig. 9a and b.

In order to estimate *E*_*radial*_, an inverse analysis was performed by varying the input Young's Modulus (*E*) in the simulation until the output stiffness value matched the experimental radial stiffness shown in Fig. 8b. The Poisson's ratio was assumed to be zero as foams such as the extruded product show negligible lateral expansion on compression (Huber and Gibson, 1988). The radial stiffness of the SD and HD tube was measured from the linear region of the experimental load-deflection data and was found to be 46 ± 11 N/mm and 53 ± 8 N/mm respectively which in turn corresponded to average *E*_*radial*_ values of 49 ± 12 MPa and 132 ± 20 MPa respectively. The numerically and analytically determined *E*_*radial*_ values of the SD and HD tubes were approximately different from each other by 24% and 14% respectively. Additionally, the stress contour plots were used as a means of determining the fracture stress in radial compression of the SD and HD tubes. At vertical displacements equal to the experimental global displacements at fracture, the horizontal stresses at the top and bottom faces of the inner diameter were extracted. These average values were found to be 1.0 ± 0.2 and 2.1 ± 0.2 MPa for the SD and HD tubes respectively, and were within the same order of magnitudes as the experimentally measured axial fracture stresses (see Table 6).

**Fig. 10:**
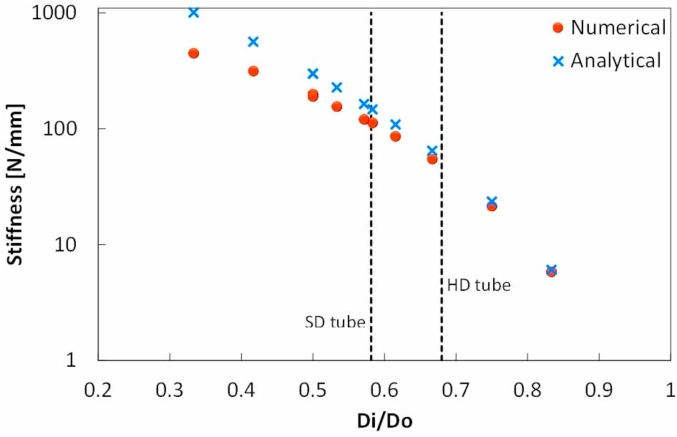
The stiffness of an annulus, with varying thickness and a fixed modulus, using an analytical thin ring solution and a numerical finite element analysis.

In order to determine the maximum thickness of the ring that the analysis is valid for, a parametric study was conducted in order to determine the stiffness of the ring by varying the ring thickness. A value for *E*_*radial*_ was arbitrarily chosen to be 120 MPa, while ring inner and outer diameters were varied between 4-11 mm and 12–16 mm respectively, to change the thickness of the ring. The stiffness (*F/δ*) gradient of the different geometries was calculated using Equation (7) and then compared to the numerical output from the FE simulations as shown in Fig. 10. As can be seen, for Di/Do ratios below 0.75, there is a divergence between the analytically calculated and numerically predicted stiffness of more than 10%. The tubes in this work lie just outside this range, and thus the numerical *E*_*radial*_ values were deemed more accurate.

In the case of the HD flatbread, a simple substitute geometry could not be used to represent the HD flatbread in radial loading and instead a solid homogenous model in the same shape as the HD flatbread was generated. Photographs of the cross-section of the HD flatbread were captured and imported into the *Solidworks CAD* software where the geometry of the specimen was created. The *Solidworks* software was subsequently used to create 3D parts which were representative of the HD extruded flatbread.

The 3D model of the HD flatbread created in *Solidworks* was imported into Abaqus as a 3D deformable solid part with linear 3D continuum solid elements. As with the FE models of the tubes, two analytical rigid parts represented the compression plates and a contact definition was applied between the outer surface of the virtual HD flatbread and the rigid bodies. A mesh sensitivity study was used to determine the optimum mesh size. An inverse analysis was performed by varying the modulus until the stiffness from the experimental tests (85 ± 25 N/mm) and numerical simulations matched, and this value was found to be 63 ± 18.5 MPa. Stress contour plots of the deformed HD flatbread are shown in Fig. 8c.

The results show that the *E*_*radial*_ values for both extruded tubes and flatbread were found to be in the same order of magnitude as their respective *E*_*axial*_ values. Thus it could be concluded that the extruded products were isotropic. The baked wafer could only be tested in the axial direction due to the nature of its geometry making it difficult to be tested in the ‘radial’ direction, however previous work which tested the wafer in flexure showed that the bending modulus was two orders of magnitude higher than its compressive modulus (Mohammed et al., 2013a). The latter is due to the dense skins of the wafer's microstructure which leads to a very stiff response in flexure. Flexural tests for the extruded products were not possible because slender beam samples could not be cut from the supplied materials.

All the results presented in this section are further discussed and compared below.

### 3.3 Comparison of the cellular products

Processing of complex multiphase systems such as the ones studied here is extremely complex due to various interacting processes occurring on different length and time scales simultaneously (Mack et al., 2013). Cereal based foams in particular are end products of several biochemical and biophysical reactions. A macro-scale description therefore of the product in terms of bulk mechanical behaviour, though useful in itself, has to be linked back to the micro-scale processes that take place such that optimisation of the manufacturing process may be achieved. The two manufacturing processes, baking and extrusion, lead to different parameters of pressure, temperature, moisture content, etc., therefore the current study compares geometrical architectures at the microscale as well as the macroscale mechanical response of the various cellular products.

The baked wafer and the extruded SD tube had a similar fracture stress (see Table 6), however the axial modulus of the SD tube was an order of magnitude higher than the baked wafer. The two products were similar in terms of porosity (see Table 3, however the microstructural makeup was different (see Table 5 and Fig. 6). Specifically, the cell wall thickness of the SD tube was similar to the values found in the wafer skin whereas the wafer core was associated with much thinner cell walls. The pore diameters in the wafer and especially in the core region were also larger than in the SD tube. Apart from the microstructural features that were measured in this study (Table 3–5) the way the material is distributed in the cell walls and faces of the structure is also important. In fact foams are often classified as open cells (all material on cell edges) or closed cells (material distributed in faces as well as edges of the cells) and this is known to have a significant effect on the global response of the foam (Gibson and Ashby, 1988). For the irregular and random cellular structures however found in foods, it is not easy to make such a distinction as both open and closed cells coexist. Chevalier et al. (2014) compared microstructures in commercial products and specifically a baked biscuit (made from leavened dough) to an extruded corn ‘ball’. They attributed marked observed differences in pore sizes and wall thickness to the distinct processing kinetics. Baking created a cellular structure progressively by the release of carbon dioxide from the leavening agent followed by water vaporisation as the temperature increased slowly in the oven, reaching 100 °C at the end of the baking stage. This was found to lead to a more open cell structure. In contrast, extruded products are commonly subjected to high temperature (above 100 °C) and pressure. At the die exit, the pressure drops and this causes an ‘explosive’ sudden expansion and a simultaneous water vaporisation. The cellular structure is therefore formed very quickly and is stabilised by immediate subsequent cooling and drying.

The two extruded tubes were produced using the same extruder. In addition, apart from the initial moisture content, the recipe was identical thus explaining why the solid density of both products was measured to be almost the same (see Table 3). In contrast, the HD tube had an overall lower porosity, a higher proportion of larger pores and thicker cell walls than the SD tube (see Fig. 6). This is in agreement with literature (Kristiawan et al., 2016) which states that for low moisture content (or high amylose content), the structure is set before bubble collapse would take place due to vapour condensation. The different microstructures of the two extruded tubes directly influenced their mechanical properties with the HD tube having both a Young's modulus and fracture stress approximately double that of the SD density tube.

The HD extruded tube and flatbread products were identical in terms of formulation however the flatbread underwent an additional rolling step after extrusion, thus altering its final geometry. Based on the images obtained from the SEM, it appeared that the rolling did not affect the microstructural features since both the HD tube and flatbread possessed pore sizes and cell wall thickness within the same range of values (see Table 4). Furthermore, the experimental density measurements showed that the relative density, ***ρ***_***relative***_, of both products were very similar (Table 3). The average mechanical properties of fracture stress, axial modulus and radial modulus values of the HD tube and the flatbread were different, however the scatter in the flatbread results was quite high and hence the differences might not be statistically different. Thus, as expected, the global product geometry did not affect the microstructure or the mechanical behaviour, i.e. rolling the extruded tube to a flatter shape before it solidified did not have an effect on its material parameters. The latter are not a function of the sample's geometry. However, this does not necessarily translate to similar sensory properties of the HD tube and HD flatbread as during oral processing, the consumer senses forces and deformations which would depend on the sample's geometry. Therefore it would be very interesting to correlate the findings of this study with sensory data for all four products.

## 4 Conclusion

Extrusion of cereal based products has potential advantages over traditional baking methods but it is hard to achieve the desired texture through this process. The evolution of the bubbly structure during processing is an extremely complex phenomenon involving various physical and chemical reactions which occur simultaneously and across a large range of time and length scales (Mack et al., 2013). In this work, new information on the impact of the manufacturing process on the cellular architecture as well as the mechanical properties of cereal based confectionery products is brought to light. Unlike empirical studies based on Texture Profile Analysis, experimental and analytical methods based on rigorous engineering analysis are outlined which enable valid and accurate comparisons of mechanical properties, taking into account the irregular geometries of the products. Quantification of such differences is crucial as it paves the way for optimising manufacturing processes.

It was shown that products manufactured through baking and extrusion led to markedly different microstructures even though the macroscopic relative densities of both materials were matched at about 0.22. Baking led to inhomogeneous skin-and-core type structures with a very weak core with larger pores and thin cell walls. In contrast, the extruded cellular structure was more homogeneous. These structural differences led to an axial compression modulus being an order of magnitude larger for the extruded product though interestingly the fracture stress in the same mode of loading was found to be similar. For the extrusion process, it was found that higher moisture content in the initial formulation led to a denser product with smaller pores and thicker cell walls. The led to a higher modulus by a factor of approximately three and a larger fracture stress by a factor of two. Finally, an effort to flatten extruded products into shapes more comparable to the shape of baked wafers, showed that the structures and mechanical properties were not affected significantly.

As the microstructure and mechanical performance of food products are thought to strongly affect sensory perception during the oral process, the work serves as a crucial step towards generating quantitative parameters to aid design and development of new confectionery snacks. Last but not least, the data reported in this study are also required as inputs to numerical predictive models which enable the separate geometrical (microstructure architecture) and material effects (cell wall properties) on the bulk response of these complex products to be decoupled.
